# The ribonuclease Dis3 is an essential regulator of the developmental transcriptome

**DOI:** 10.1186/1471-2164-13-359

**Published:** 2012-08-01

**Authors:** Dezhi Hou, Miriam Ruiz, Erik D Andrulis

**Affiliations:** 1 Department of Molecular Biology and Microbiology, Case Western Reserve University School of Medicine, Wood Building, W212, Cleveland, OH, 44106, USA

**Keywords:** Dis3, Ribonuclease, RNase, *Drosophila melanogaster*, RNA metabolism, RNA turnover

## Abstract

**Background:**

Dis3 is ribonuclease that acts directly in the processing, turnover, and surveillance of a large number of distinct RNA species. Evolutionarily conserved from eubacteria to eukaryotes and a crucial component of the RNA processing exosome, Dis3 has been shown to be essential in yeast and fly S2 cells. However, it is not known whether Dis3 has essential functions in a metazoan. This study inquires whether Dis3 is required for *Drosophila* development and viability and how Dis3 regulates the transcriptome in the developing fly.

**Results:**

Using transgenic flies, we show that Dis3 knock down (Dis3KD) retards growth, induces melanotic tumor formation, and ultimately results in 2^nd^ instar larval lethality. In order to determine whether Dis3KD fly phenotypes were a consequence of disrupting developmentally regulated RNA turnover, we performed RNA deep sequencing analysis on total RNA isolated from developmentally staged animals. Bioinformatic analysis of transcripts from Dis3KD flies reveals substantial transcriptomic changes, most notably down-regulation in early expressed RNAs. Finally, gene ontology analysis of this early stage shows that Dis3 regulates transcripts related to extracellular structure and remodelling, neurogenesis, and nucleotide metabolism.

**Conclusions:**

We conclude that Dis3 is essential for early *Drosophila melanogaster* development and has specific and important stage-specific roles in regulating RNA metabolism. In showing for the first time that Dis3 is required for the development of a multicellular organism, our work provides mechanistic insight into how Dis3—either independent of or associated with the RNA processing exosome—participates in cell type-specific RNA turnover in metazoan development.

## Background

*Drosophila melanogaster* development requires the precise coordination of multiple distinct gene regulatory mechanisms and processes within, between, and among different cell types. One such process, RNA turnover, ensures that free nucleotides are salvageable for use in transcription, signalling, transport, and protein translation. RNA turnover is especially important during cellularization, when all maternally deposited RNAs are degraded [1]. Yet, surprisingly, the full set of ribonucleases (RNases) and RNA-binding proteins that contribute to developmentally regulated RNA turnover—both maternal and zygotic RNAs—remain unknown.

Dis3—a 3’ to 5’ exoRNase and endoRNase—has vital, conserved roles in RNA turnover and surveillance in eukaryotic cells [2-4]. A homolog of the prokaryotic RNase II and RNase R [5], Dis3 has been proposed to be the major ribonucleolytic activity in the RNA processing exosome [6], a protein complex consisting of the nuclear 3’ to 5’ exoribonuclease Rrp6, RNase PH subunits Rrp41/Ski6, Rrp42, Rrp43, Rrp45, Rrp46 and Mtr3, and S1 domain subunits Rrp4, Rrp40 and Csl4 [7,8]. Although functions of the Dis3 RNase have been attributed to the exosome, we and others have proposed that Dis3 and exosome subunits may individually assemble into and/or function in exosome-independent complexes [9-11]; we call these complexes “exozymes [12].” One such exozyme is a complex of Dis3 and Rrp6 with Importin-α3, although its function remains unclear [13]. In this regard, Dis3 and Rrp6—but no other exosome subunits—have roles in the cell cycle, presumably related to their core exosome-independent RNA substrates and activities [11,14,15]. Finally, Dis3, Rrp6, and the core exosome play non-overlapping roles in rRNA, mRNA, tRNA, and other RNA species metabolism [2,3,16].

Despite progress towards understanding Dis3 substrates and activities in an individual cell, we know nothing of its contributions to RNA metabolism during development of a multicellular organism. This is a fundamental issue in need of clarification, as spatiotemporal control of RNA deposition, expression, and turnover are central to proper ontogenesis [17-19]. Supporting a role for Dis3 in development, Dis3 mRNA is present in almost all cells in the *Drosophila* embryo and Dis3 protein is detectable at every stage of *Drosophila* development [20]. Further support comes from microarray data showing that Dis3 depletion affects expression of developmental and neuronal transcripts in embryo-derived tissue culture cells [21].

Given that *Drosophila* development and transcriptomics are well-characterized, and that the fly is a tractable genetic system, we set out to study the role of Dis3 in RNA metabolism during ontogenesis using transgenic knock down fly strains. By analyzing the appearance of staged Dis3-depleted flies, the cytology of isolated fly organs, and the expression and pathways of total and specific RNAs, we provide the first evidence that Dis3 has an essential role in a metazoan.

## Results

### Generation of Dis3 knock down flies

**Figure 1:**
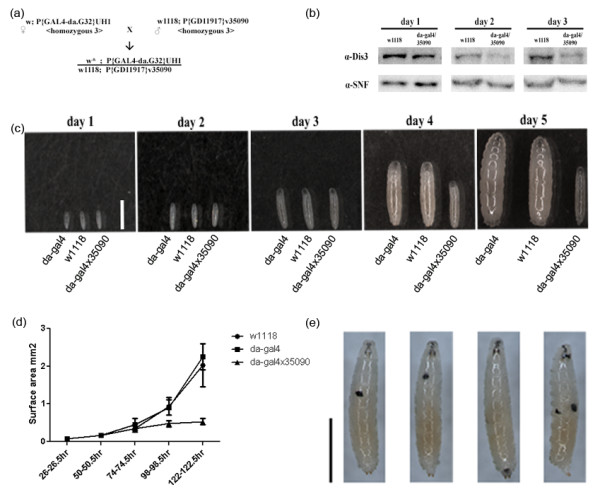
**Dis3 is essential for fly development. (a)** Scheme for generating Dis3 RNAi flies with Gal4/UAS-driven transgenic RNAi system. **(b)** Western blots on nuclear extracts show decrease of Dis3 protein level in Dis3 knock down flies at different larval stages; SNF is used as a loading control. **(c)** Larval growth of da-Gal4 (left), w1118 (middle) and da-Gal4 x 35090 (right) strain; white bar = 1 mm. **(d)** Growth curve of da-Gal4, w1118, and da-Gal4 × 35090 fly larvae measured by surface area (mm^2^). Error bar was standard deviation of measurement of ≥ 30 larva samples. **(e)** Melanotic masses in da-Gal4 × 35090 fly larvae; black bar = 1 mm.

Working in the *Drosophila melanogaster* S2 tissue culture system, our group showed that the Dis3 RNase is essential for growth and for proper RNA metabolism [21]. We also showed that Dis3 regulated a set of RNAs that were functionally related to developmental processes. Because no study has been attempted to understand the role of Dis3 in development, we set out to address this shortcoming. To this end, we crossed a fly strain harboring a daughterless-Gal4 (da-Gal4) driver to a strain with a UAS promoter driving a Dis3 RNAi transgene, thereby generating several Dis3KD transgenic flies (Figure 1a, Table 1). Following the cross, larvae were harvested at three different days to determine the level of Dis3 protein depletion. A comparison of the wild type control flies (w1118) to the Dis3 RNAi flies (da-Gal4/35090) revealed that Dis3 protein level was reduced in all three different larval stages, with greatest amount of protein depletion on the 3^rd^ day (Figure 1b). We used this transgenic system to address the effects of Dis3 depletion on fly development.

**Table 1:** Fly strain genotypes and phenotypes

**Strain**	**Growth rate**	**masses**	**terminal phenotype**
w1118	+++	-	viable
da-Gal4	+++	-	viable
tub-Gal4	+++	-	homozygous lethal
act5c-Gal4	+++	-	homozygous lethal
UAS-Dis3 (35090)	+++	-	viable
UAS-Dis3 (35091)	+++	-	homozygous lethal
da-Gal4/UAS-Dis3 (35090)	+	+	2nd instar larva lethal
da-Gal4/UAS-Dis3 (35091)	+	+	2nd instar larva lethal
tub-Gal4/UAS-Dis3 (35090)	+	+	2nd instar larva lethal
act5c-Gal4/UAS-Dis3 (35090)	+	+	2nd instar larva lethal

### Dis3 knock down larvae are growth retarded and 2^nd^ instar lethal

We first sought to determine whether Dis3 depletion had any overt effects on embryo morphology or developmental timing. We isolated and examined individual embryos and larvae from control w1118, da-Gal4, and da-Gal4/35090 flies over 5 days (Figure 1c-d). Whereas the control animals entered a period of rapid growth during the transition from the 3^rd^ to 5^th^ day, the da-Gal4/35090 animals slowed down: 477% and 396% growth for the w1118 and da-Gal4 flies, respectively, and 50% growth for the da-Gal4/35090 flies. Further, the da-Gal4/35090 flies stay as 2^nd^ instar larvae for two weeks prior to exhibiting 100% lethality (Table 1). Most of the da-Gal4/35090 larvae have one or more melanotic masses that are distributed throughout the organism (Figure 1e). As these masses are cell nodules that arise due to inappropriate signalling during hematopoeisis [22], these data indicate that proper Dis3 levels are required for blood cell function and differentiation during development.

In order to confirm these phenotypes, we performed crosses with another Dis3 RNAi strain (35091) and with other Gal4 driver strains like tub-Gal4 and act5c-Gal4. We examined larval growth, melanotic masses, and lethality of these crossed strains (Table 1). All of the Dis3KD flies exhibited the same phenotypes, confirming our initial results (Figure 1). Based upon this finding and as the da-Gal4 driver has been shown to express ubiquitously throughout development [23], we performed all subsequent analyses with the da-Gal4/35090 Dis3KD flies and w1118 wild-type (WT) control flies.

### Dis3 knock down does not affect fly brain morphology

**Figure 2:**
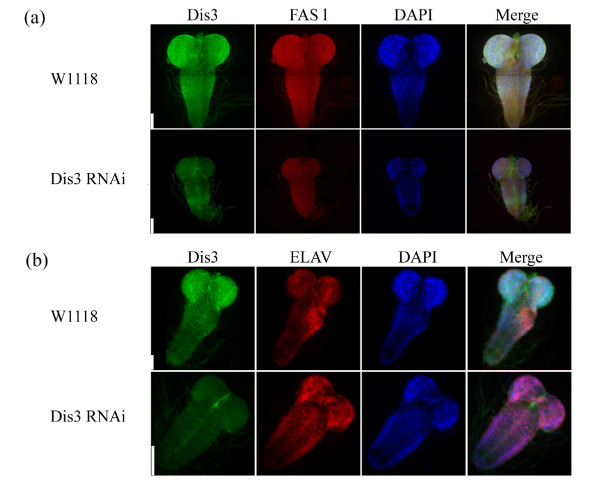
**Dis3 depletion affects brain growth but does not impact neuronal marker protein distribution or levels.** Wild type w1118 and Dis3 RNAi fly larvae were collected at day 5, brains were dissected and stained with **(a)** anti-Dis3 (green), anti-fasciclin (FAS1; red) or with **(b)** anti-Dis3 (green) and anti-ELAV (red). Nuclei were stained with DAPI (blue).

In our prior microarray study, we discovered several enriched Dis3 target RNAs that were related to neurogenesis [21]. We predicted that if Dis3 were regulating these RNAs during development, we should find Dis3 localizing to fly brains. To test this prediction, we dissected whole brains from WT and Dis3KD larvae and co-stained them with antibodies to Dis3 and the neuronal marker protein fasciclin (Figure 2a), a microarray-identified Dis3 target RNA. In the WT brain, both anti-Dis3 and -fasciclin antibodies stained the whole organ; these staining patterns appeared to overlap with one another. A close-up examination of anti-Dis3 antibody co-stain with DAPI (Additional file 1: Figure S1b) reveals neuron-specific staining that is either cytoplasmic or nuclear; this compartment exclusivity was also seen in embryonic tissue culture cells [10,13]. Although the Dis3KD fly brains are half the size of WT brains, we did not detect any otherwise aberrant morphology; we also did not observe changes in anti-fasciclin antibody staining in Dis3KD brains. Nonetheless, we detect Dis3 depletion as loss of anti-Dis3 antibody staining (Figure 2a), supporting the depletion observed with our western blotting results (Figure 1b).

We sought to use indirect immunofluorescence as an indirect test of whether Dis3 depletion affected general mRNA metabolic pathways in brains. To this end, we explored the protein localization and levels of the neuron-specific mRNA-binding factor ELAV ([24]; Figure 2b). In WT brains, anti-Dis3 and -ELAV antibodies exhibited non-overlapping staining patterns. In Dis3KD brains, both the anti-ELAV antibody staining pattern and signal level were largely unaffected. Our data thus suggest that Dis3KD fly phenotypes are not a by-product of perturbing the localization and levels of proteins in general mRNA metabolic pathways.

In prior work, we showed that Dis3 and Rrp6 physically interact and co-localize in S2 cells and are mutually required for proper localization [13]. To determine whether these protein partners co-localize and cooperate in flies, we stained WT fly brains with antibodies to Rrp6 and Dis3. Surprisingly, anti-Rrp6 antibodies do not stain the brain lobes, whereas anti-Dis3 antibodies do; anti-Rrp6 antibodies stain certain brainstem portions, but this staining is not found in all brain stains (Additional file 1: Figure S1a; data not shown). Further, Dis3 depletion did not significantly affect the anti-Rrp6 antibody staining pattern. These observations suggest that Dis3 and Rrp6 may not cooperate in all *Drosophila* tissues, consistent with the exozyme hypothesis [12].

### Transcriptomic profiling of Dis3 knock down flies

**Figure 3:**
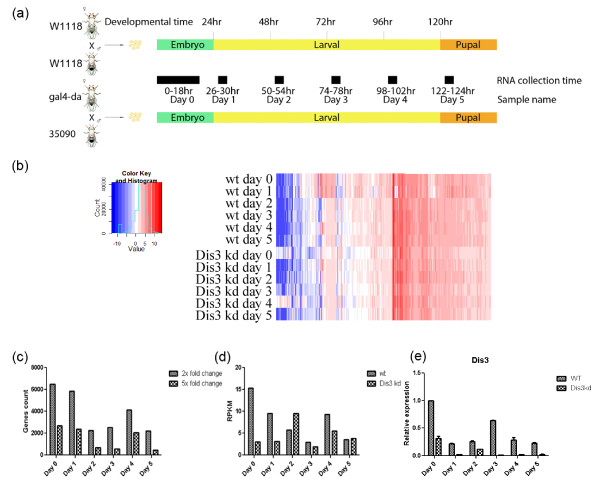
**Transcriptomic analysis of Dis3 knock down flies. (a)** Experimental design; black bar indicates the RNA collection time for specific development time periods. **(b)** Heatmap shows the genome-wide RNA expression profile. Rows correspond to the wild-type (w1118) and Dis3-depleted (Dis3KD) development stages; columns correspond to 11,665 annotated *Drosophila* genes. The color key represents log_2_ transformed RPKM value. Red indicates high expression; blue indicates low expression. Expression profiles are clustered using hierarchical clustering. **(c)** Number of RNAs with 2x fold change and 5x fold change in Dis3KD flies in 6 development time points as compared to wild type files. **(d)** Dis3 relative expression level in wild type and Dis3KD flies in 6 development time points. **(e)** Relative Dis3 expression level in wild type and Dis3KD flies in 6 development time points measured by qRT-PCR.

Given the role of Dis3 in regulating a defined subset of the S2 cell transcriptome, we hypothesized that Dis3 depletion affects fly development by perturbing either the expression, processing, and/or turnover of vital developmental transcripts. To test this hypothesis in an unbiased and thorough manner, we performed RNA deep sequencing (RNA-seq) analysis of WT and Dis3KD flies during development. To capture snapshots of the fly transcriptome at specific developmental stages, we divided our analysis into 6 time points (Figure 3a). At the first time point (day 0), embryos were collected after flies laid eggs for 18 hours. For all other time points (days 1 to 5), the flies laid eggs for 4 hours and samples were collected after 26, 50, 74, 98 and 122 hours. We collected WT and Dis3KD flies in parallel to permit comparison.

Following RNA extraction, purification, preparation, and deep sequencing, the raw RNA-seq data was processed, quantified, and normalized, and RPKM values were calculated (Additional file 2: Table S1 and Additional file 3: Table S2). From this analysis, a total of 14,623 transcripts were mapped to the *Drosophila* genome, including 19 new, previously unannotated genes. Of these transcripts, the 11,665 that had high raw read count (> 20) in at least one sample were selected for further analysis. To organize those transcripts, we generated a heatmap with the log_2_-transformed RPKM values for every time point (Figure 3b).

Our heatmap revealed a specific RNA accumulation pattern in day 0 and day 4 Dis3KD samples as compared to the WT samples (Figure 3b). We isolated this transcript subset and generated a detailed heatmap (Additional file 4: Figure S2a). To determine the nature of these effects, we performed a gene ontology (GO) enrichment study. In three GO categories—“biological process,” “cellular components,” and “molecular function”—we selected the five GO terms with the top P-value scores and then graphed them by both number of transcripts and fold enrichment (Additional file 4: Figure S2b). The highest scoring GO terms in the Dis3KD data set correspond to “biometabolism of metabolites,” “chemical energy,” “mitochondria,” and “membrane transporters.” Notably, these GO terms are unified in the phenomenon of oxidative phosphorylation, suggesting that Dis3 may be involved in this process.

### Dis3 knock down affects early transcriptomic expression

In order to examine the broader impact of Dis3 depletion on the *Drosophila* transcriptome, we graphed the numbers of both increased and decreased transcripts. (We applied a filter to eliminate transcripts with low expression in both samples.) These transcripts were grouped as 2 fold or greater change (2x) and 5 fold or greater change (5x) for each time point (Figure 3c; Additional file 5: Table S3 and Additional file 6: Table S4; the 2x data set includes the 5x set within itself). Dis3KD affected the largest number of RNAs at early time points, with 55.8% of the transcriptome affected in day 0 and 50.1% in day 1 (Figure 3c). At later time points, a smaller portion of the transcriptome is affected, with ~22% in days 2, 3, and 5 and 35% on day 4; this greater effect in day 4 was already intimated (Additional file 4: Figure S2).

In order to examine whether Dis3 expression correlated with these stage-specific effects, we extracted the Dis3 expression data from RNA-seq for WT and RNAi-depleted animals. We find that Dis3 transcriptional pattern undulates from high expression during early embryogenesis to low levels prior to late stages of larval development (Figure 3d). Consistent with expectations, Dis3KD elicits reduction of Dis3 RNA (Figure 3d); this reduction was further validated using quantitative real-time RT-PCR with actin as an unaffected loading control (Figure 3e; qRT-PCR). Together, the correlation between Dis3 RNA levels and depletion with robust transcriptomic effects at early time points supports an important role for Dis3 in RNA metabolism during early developmental stages.

Expanding upon the initial fold change analysis (Figure 3c), we graphed the number of 2-fold and 5-fold increased and decreased RNAs at each time point in Dis3KD samples (Additional file 7: Figure S3). We find that on days 0 and 1, RNAs are predominantly decreased. In contrast, for day two through day five, we find equivalent numbers of increased and decreased RNAs.

### Gene ontology analysis of transcriptomic changes due to Dis3 knock down

**Figure 4:**
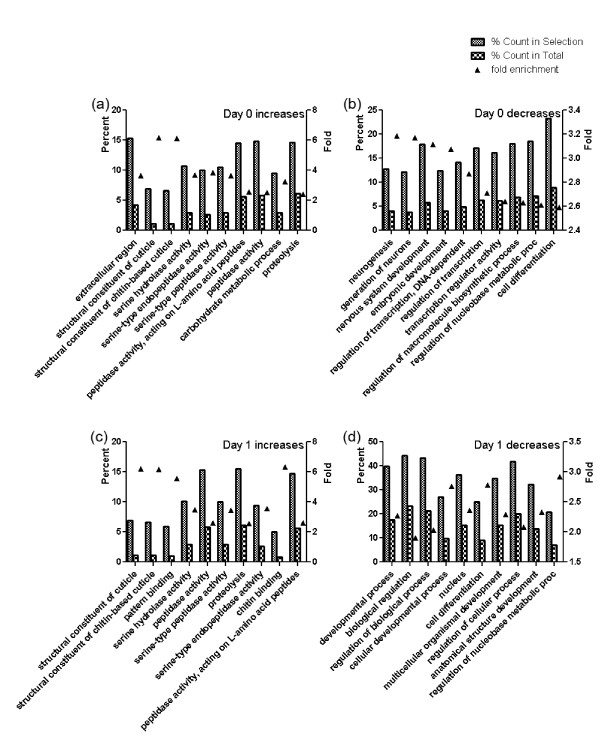
**Select gene ontology enrichment analyses of RNAs in early development.** Column charts represent the percentage of GO term genes in both the 5-fold increased and decreased RNA pools and in the whole genome RNA pools. Day 0 and 1 increases are shown in **(a)** and **(c)** whereas decreases are shown in **(b)** and **(d)**. Black triangle represent enrichment fold which was plotted on the right Y axis and calculated by dividing the % count in selection by % count in total. The 10 GO terms are sorted and selected based upon the highest P-values.

In order to determine whether there is any functional specificity for Dis3-mediated regulation during development, we performed GO analysis on those RNAs that were 5-fold increased or decreased in Dis3KD samples (Figures 4 and Additional file 8: Figure S4; Additional file 9: Table S5). For that pool of RNAs, we restricted our analysis to the top 10 GO terms for each time point as judged by their P-values. For the increased RNAs during the first two days of our Dis3KD developmental time course, enriched GO terms encompass phenomena related to cell structure and remodelling (Figure 4a-c); for the last four days, the upregulated transcripts share GO terms related to extracellular sensing, stress, and metabolism (Additional file 8: Figure S4e-h). For the decreased RNAs over the first two days of our Dis3KD developmental time course, the enriched GO terms correspond to development and differentiation as well as nucleotide metabolism (Figure 4b-d); for the last four days of our time course, the down-regulated transcripts share GO terms related to cell-cell signalling, transmembrane and channel activity (Additional file 8: Figure S4a-d). Although there is no unifying GO term that defines a single time point, our data reveal that Dis3 depletion causes specific effects on discrete classes of transcripts and pathways at different stages of *Drosophila* development.

### Dis3 downregulates early expressed RNAs during development

**Figure 5:**
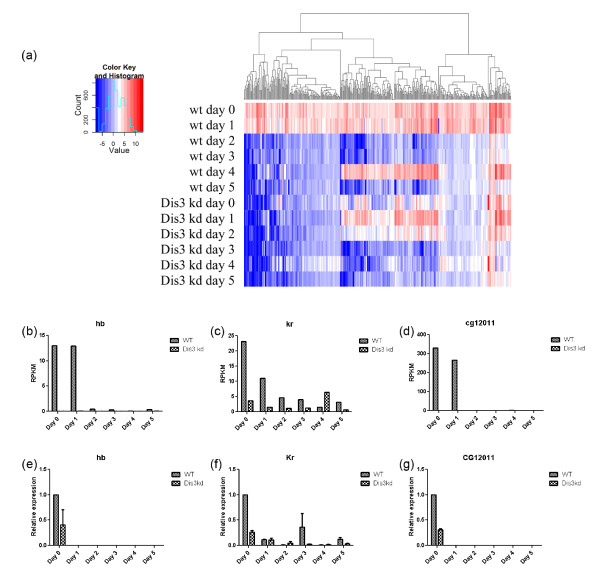
**Dis3 knock down has a pronounced effect on the accumulation of a large number of early developmentally-expressed RNAs. (a)** Heatmap of all early expressed RNAs throughout the development in wild type and Dis3KD flies. Phylogenetic analysis presented above the columns reveals how the affected RNAs in the Dis3KD sample are clearly grouped. RNA-seq derived relative expression level of **(b)** hunchback, **(c)** Krüppel, and **(d)** CG12011 during development of WT and Dis3KD flies. Real-time quantitative PCR analysis of **(e)** hunchback, (f) Krüppel, and **(g)** CG12011 at the same developmental time points.

To answer the question—How does Dis3 depletion disrupt developmental timing?—we examined early expressed RNAs in our raw RNA-seq data sets. We isolated the 514 RNAs in the WT flies that are expressed at very high levels in day 0 and day 1 but decreased significantly thereafter (~10 fold decrease cut off). We then organized and presented these RNAs as a heatmap for both the WT and Dis3KD flies over our time course (Figure 5a). We find two distinct effects of Dis3KD on these early RNAs. First, greater than 50% of the early expressed RNAs were robustly downregulated in Dis3KD flies in days 0 and 1. Second, those RNAs that showed similar expression between the WT and Dis3KD flies in days 0 and 1 persisted at high expression at day 2 only in the Dis3KD flies. We also find a striking effect when comparing these early-expressed transcripts on day 4: one-third of the transcripts that are highly upregulated in the WT (the larval-pupal transition, Figure 3a) are highly downregulated in the Dis3KD flies. Together, these data provide strong evidence for Dis3 transcriptomic regulation in the embryo, at embryonic-larval transition, and at the larval-pupal transition.

To further examine confirm our RNA-seq data, we selected early expressed RNAs from our data set for graphical analysis. Two of these, hunchback (Figure 5b) and Krüppel (Figure 5c), encode DNA-binding proteins that are known to be present in the early embryo [25]. The third RNA is annotated but has no known function: CG12011 (Figure 5d). In WT flies, these transcripts express at the first 2 time points. In Dis3KD flies, these three RNAs are substantially reduced at these early time points. To independently validate the early expression of these RNAs and the Dis3KD effects seen by RNA-seq, we performed qRT-PCR with actin as a loading control (Figure 5e-g). The general trends are largely similar, with RNAs detected at early time points and Dis3KD eliciting their reduction. We suspect the differences between qRT-PCR and RNA-seq arise from the nature of RNA preparation and from the manner and efficiency of sequence detection and amplification. Finally, we verified that the changes in hunchback, Krüppel, and CG12011 mRNA levels were not observed in the da-Gal4 early embryo (Additional file 10: Figure S5).

### Analysis of exosome subunits expression during Drosophila development

Given the established role of Dis3 in the RNA processing exosome—and given that the exosome has vital roles in numerous RNA metabolic pathways—we considered the possibility that the Dis3KD changes in the developmental transcriptome might arise from perturbation of exosome subunit RNA expression. To test this hypothesis, we isolated and graphically analysed the RNA-seq-determined expression of Rrp6 and core exosome subunits (Additional file 11: Figure S6). While Dis3KD elicits a significant knockdown of Rrp6 RNA levels at day 0 and 1, there is no measurable effect at later developmental time points (Additional file 11: Figure S6a). We see a similar pattern (but not as strong as the early effects on Rrp6) of Dis3KD-mediated effects on RNase PH and S1 subunits as well (Additional file 11: Figures S6b-i), with a few subunit RNAs showing decrease levels at the day 4 time point. These data suggest that Dis3KD effects on early RNA metabolism may be accentuated by impacting the expression of Rrp6 or exosome subunit RNAs.

## Discussion

In this study, we examine the mechanistic contributions of Dis3—an evolutionarily conserved ribonuclease and a component of the major RNA metabolic complex, the exosome—to *Drosophila development*. Using RNAi to deplete Dis3 RNA, we demonstrate that Dis3 is essential in a metazoan. We identify and categorize Dis3 target RNAs using RNA-seq and reveal specific classes of RNAs that are impacted at discrete developmental periods. We observe both the highest number of affected RNAs and the greatest changes in RNA expression in the embryonic and first instar larval points, indicating that Dis3 plays important roles in regulating the early *Drosophila* transcriptome.

When Dis3 is depleted, flies grow more slowly, have a reduced body size in the second instar, die with smaller brains, and accumulate melanotic masses. We interpret and unify these phenotypes as a role for Dis3 in regulating proper timing of cell cycle progression in a multi-cellular organism; that is, when Dis3 is functionally perturbed, the cell cycle is delayed. Prior work in fission yeast supports this idea, as mutation in Dis3 leads to aneuploidy and defects in passage through mitosis [26-28]. Further, we recently showed that Dis3 disrupts timing of spindle formation and positioning and perturbs RNA metabolism of critical cell cycle stage-specific RNAs in budding yeast [15]. Although it has been proposed that the Dis3 ribonuclease activity is required for mitotic progression, the RNase domain mutant used in that study retains enzymatic activity [14], perhaps due to its endonuclease activity. As we still detect Dis3 protein in depleted flies by both western blotting and immunofluorescence, we suggest that our phenotypes (and those in prior studies) are due to diminished substrate recognition and metabolism rather than loss of RNase activity *per se*. We hypothesize that the ultimate phenotypic consequence of reduced Dis3 expression is the melanotic masses, a characteristic of defective blood cell homeostasis and development [29-31]. On this note, the closest human homolog to Dis3 is located at 13q21, a chromosomal locus linked to a variety of cancers, including lymphocytic leukemia [32,33]. Further, mutations in another human homolog, Dis3L2, have been recently shown to cause the Perlman syndrome of overgrowth and Wilms tumor susceptibility in the germline [34]. The exact mechanism by which Dis3 perturbation elicits melanotic masses in flies is thus clearly of interest as it may be a potential model for understanding blood cell regulation specifically and tumorigenesis generally.

Our work shows that Dis3 has a prominent role in regulation of the early *Drosophila* transcriptome. For example, Dis3KD affects higher levels of RNAs and shows a greater range of effects (fold effects that are both increased and decreased) at early time points as opposed to later ones. Moreover, we find that Dis3KD downregulates known early-expressed RNAs in particular. Because we initially expected that Dis3 depletion would lead to more upregulated RNAs, we interpret this transcriptomic downregulation to mean that Dis3 inhibits with and/or out-competes other ribonucleases to maintain proper RNA and nucleotide levels. For example, in the absence of Dis3, other RNases, such as Rrp6 or the exosome, may become more active. Given the surveillance roles for Rrp6 in both yeast [35] and *Drosophila*[36], this is a possibility; this turnover could be post- or co-transcriptional, as *Drosophila* Rrp6 and the exosome occupy transcriptionally active genes [37]. Another possibility is that Dis3 may affect an mRNA encoding a global transcriptional repressor, thus indirectly downregulating the transcriptome. An alternative interpretation—predicted by a systems theory that explicates the flow of genetic information as nested cycles [38]—is that the transcription cycle is sensitive to changes in nucleotide levels, and, in disrupting RNA turnover, the transcription cycle slows down, ultimately affecting all supervenient cycles, especially the cell cycle. Supporting this interpretation, genetic and nutrient changes that affect cell cycle timing also throw off yeast transcriptomic cycle timing [39]. Unfortunately, our time points do not permit discrimination between effects on maternally deposited RNAs and those on zygotic transcription. Nonetheless, because Dis3 has such pronounced effects on early RNA stability, future studies that explore its activities during cellularization will be important to clarify our findings here.

## Conclusions

We show that Dis3 is essential for proper transcriptomic regulation during *Drosophila* development. In this regard, this work importantly builds upon our general understanding of the regulators of—and transcriptomic changes that occur during—*Drosophila melanogaster* development [40-43]. Finally, this study sets the stage for future analyses to understand the precise contributions of Dis3 and other ribonucleolytic enzymes to RNA metabolic pathways and gene expression during metazoan development.

## Methods

### Fly strain and crosses

Flies were raised on standard cornmeal and agar media at room temperature. Wild type strain W1118 and UAS-Dis3 RNAi strain v35090 (w^1118^; P{GD11917} v35090, Flybase ID: FBst0460972) and v35091 (w^1118^; P{GD11917} v35091/TM3, Flybase ID: FBst0460973) were obtained from Vienna Drosophila RNAi Center (VDRC: http://stockcenter.vdrc.at). The Gal4 driver lines act5c-Gal4 (y[1]w[*]; P{Act5C-GAL4-w-}E1/CyO), da-Gal4 (w*; P{Gal4-da}) and tub-Gal4 (yw*; P{w^+mc^:tubP-Gal4}LL7/TM3, Sb) were obtained from Bloomington Drosophila Stock Center (http://flystocks.bio.indiana.edu/). To knock down Dis3 mRNA in flies, males of UAS-Dis3 RNAi strains were crossed to virgin females of Gal4 driver lines. Embryos were collected at room temperature on grape plates for a time period as experiment required. Larvae were transferred to new vials and grown at room temperature.

### Larval measurement and analysis

From larval size measurements, ~40 larvae were collected at each time point and images were captured with a digital camera. We imported the images into Adobe Photoshop and measured the larval surface areas by setting the scale to count pixels and then converted them into metric units. Surface area was calculated in Microsoft Excel and plotted in Graphpad Prism.

### Western blotting

Fly larvae were collected, frozen in liquid N_2_ and crushed into powder, then resuspended in buffer I (15mM HEPES pH 7.4/5mM KCl/5mM MgCl2/0.2mM EDTA/350mM sucrose/1mM DTT) supplemented with protease inhibitor cocktail (PIC; Roche). The sample was homogenized, run through a syringe, and centrifuged at 6,000 x g for 15 mins. Supernatant was collected as cytosolic extract and the pellet was washed and lysed with buffer II (50mM HEPES pH 7.6/110mM KCl/250mM NaCl/0.1mM EDTA/0.1% Tween-20/10% glycerol/1mM DTT) with PIC, on ice, for 15 mins. Nuclear extract was collected by centrifuge at 9400 x g for 20min, 1 volume 2x SDS loading buffer was added, and then boiled for 5min at 95°C. Western blotting was performed as described previously. Anti-Dis3 and anti-SNF antibodies were used 1:1000.

### Immunostaining

Larvae were collected at day 5, brains were dissected under a light microscope and placed in ice cold PBSS (PBS + 0.02% Saponin). Brains were fixed in PBSS with 4% formaldehyde for 20 min at room temperature, washed, then blocked with freshly made 5% NDS (Normal Donkey Serum diluted in PBSS) and followed by antibody and DAPI staining as described [44]. Anti-Dis3, anti-Fasciclin, anti-ELAV and anti-Rrp6 were used at 1:1000, 1:500, 1:500, and 1:1000 respectively. The CY2- or Texas red-conjugated secondary antibodies were used at 1:500. Stained brains were mounted and imaging was carried out using a Zeiss microscope with a 40x objective.

### RNA collection and RNA deep sequencing

For day 0 samples, embryos were collected after 18 hr egg laying; for later time points, flies laid eggs for 4 hrs and the larvae were collected at 24 hr intervals, every day for 5 days. At each time point, a total of 50 mg embryos or larvae were collected and frozen, total RNA was isolated using Trizol (Invitrogen), treated with DNase, and passed over a column (RNeasy kit; Qiagen) then sent to Microarray and Genomic Analysis Core Facility of the Huntsman Cancer Institute (University of Utah). RNA libraries were generated at the core facility using Illumina TruSeq RNA sample prep kits. Six libraries were sequenced (50 cycles) simultaneously in a single lane of an Illumina HiSeq 2000.

### Data analysis

A sequencing file for each individual sample was uploaded in to the Galaxy website (http://main.g2.bx.psu.edu/). Raw reads were groomed with FASTQ groomer and aligned to *Drosophila* reference genome (dm3) with Tophat for Illumina. Files were then uploaded into Avadis NGS software, where quantification and normalization were performed. The RPKM value for each gene were calculated and used for a relative gene expression, following which fold change and gene ontology analysis were performed. The heatmap of the whole genome and subset genes were generated in R (version 2.13) with heatmap.2 function that is included in gplots library. DAVID 6.7 (http://david.abcc.ncifcrf.gov/) was used to analyze the gene ontology of subset genes highlighted in the heatmap. All the bar charts and dot plots in the analysis were done in Graphpad Prism.

### Quantitative real-time RT-PCR

The total RNA of the 12 fly samples left over from RNA-seq was used for qRT-PCR analysis. First-strand cDNA synthesis was performed with the Quantitect Reverse Transcription kit (Qiagen), according to the manufacturer’s instructions; 1 μg total RNA from each fly sample was used for cDNA synthesis. Quantitative real-time PCR (qPCR) was carried with SYBR Green PCR master mix (Qiagen) and BIO-RAD iCycler IQ real-time PCR system. The following gene-specific primers were custom designed using Primer3 with the full mRNA sequence of each gene and synthesized by Eurofins: Actin 5c (act5c): forward primer, 5’-AGTTGCTGCTCTGGTTGTCG-3’; reverse primer, 5’-CGTAGGACTTCTCCAACGAGG-3’. Dis3: forward primer, 5’-GGCCAAGGATGAAAATGAGA-3’; reverse primer, 5’-CACAAAGATGTGCCGATTTG-3’. Hunchback (hb): forward primer, 5’-CCTTACGAAAATCCCGACAA-3’; reverse primer, 5’-GATGATGACCTGGCTCCTGT-3’. Krüppel (kr): forward primer, 5’-CCTTTAGGTAGTGGCAAGCAC-3’; reverse primer, 5’- GCTGATCTCGGTCTGAAACTC -3’CG12011: forward primer, 5’-GAGGTCAAGCAG-GAAGTTGC-3’; reverse primer, 5’-TGGACATCAGGAGTTGTGGA-3’. The relative expression level of each gene was calculated for each development time points relative to the first time point.

## Competing interests

The authors declare that they have no competing interests.

## Authors’ contributions

EA and MR conceived the project; DH, MR, and EA designed the experiments; MR and DH performed fly crosses, culture, isolation, dissection and microscopic analysis, staged animals and isolated RNA; DH performed the qRT-PCR and bioinformatic, computational, and comparative analysis of the RNA-sequencing data; DH and EA wrote the paper, compiled and arranged figures, legends, and tables. All authors read and approved the final manuscript.

## Supplementary Material

Additional file 1 **Figure S1.** Dis3 depletion has little or no effect on Rrp6 localization in the fly brain. (a) Wild-type w1118 and Dis3 RNAi brains were dissected and stained with anti-Dis3 (green) and anti-Rrp6 (red) antibodies. Nuclei were stained with DAPI (blue). (b) Close-up visualization of anti-Dis3 antibody and DAPI staining of brains. Click here for file

Additional file 2 **Table S1.** Raw RNA-seq data. Click here for file

Additional file 3 **Table S2.** Filtered RNA-seq data. Click here for file

Additional file 4 **Figure S2.** A subset of mitochondria- and nucleotide-related RNAs highly up-regulated in day 0 and day 4 Dis3KD flies. (a) Comparative heatmap shows the 850 up-regulated RNAs in the Dis3KD flies. (b) GO analysis of the 850 increased transcripts reveals the enriched subset. Click here for file

Additional file 5 **Table S3.** 2-fold affected RNAs. Click here for file

Additional file 6 **Table S4.** 5-fold affected RNAs. Click here for file

Additional file 7 **Figure S3.** Detail fold change analysis. 2 fold- and 5 fold-increased and decreased RNAs in Dis3KD samples in all 6 developmental stages. This figure complements Figure 3c. Click here for file

Additional file 8 **Figure S4.** Gene ontology enrichment analyses of 5 fold-increased and -decreased RNAs for the later developmental time points. Bars depict the percentage of RNAs in selected GO term; black triangles represent fold enrichment, plotted on the right Y axis and calculated by dividing the % count in selection by % count in total. GO terms are sorted by P-value and top 10 were picked at each time point. This figure complements Figure 4. Click here for file

Additional file 9 **Table S5.** Gene ontology analysis. Click here for file

Additional file 10 **Figure S5.** Transcriptomic changes observed in the Dis3KD flies are not detected in the daughterless-Gal4 early embryos. Expression of hunchback (hb), Krüppel (kr), and CG12011 was assessed by qRT-PCR. Click here for file

Additional file 11 **Figure S6.** Relative expression level of Rrp6 and exosome subunit RNAs in wild type and Dis3KD flies over developmental time course. (a) Rrp6, (b) Csl4, (c) Rrp4, (d) Rrp40, (e) Mtr3, (f) Ski6, (g) Rrp46, (h) Rrp45, and (i) Rrp46. Click here for file
