# Supplementary figures and images for: The ribonuclease Dis3 is an essential regulator of the developmental transcriptome
Source: BMC Genomics. 2012 Aug 1;13:359. doi: 10.1186/1471-2164-13-359 (PMC3434026; doi:10.1186/1471-2164-13-359)

(a)

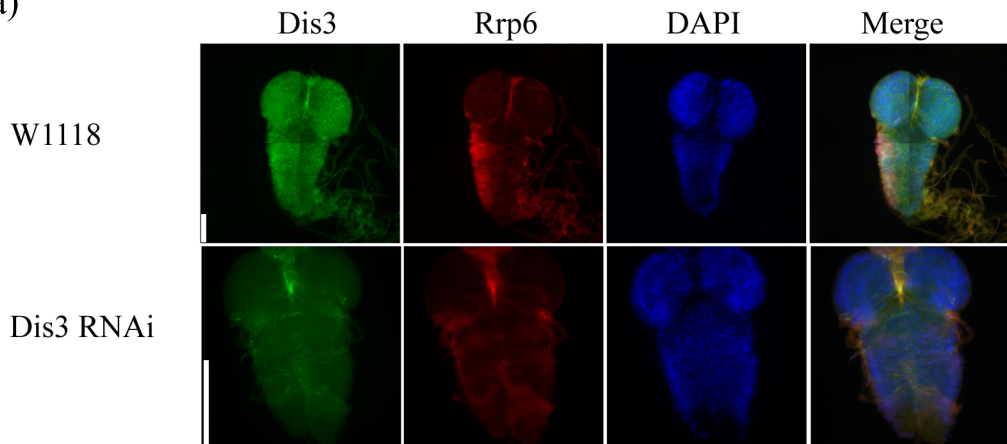

(b)

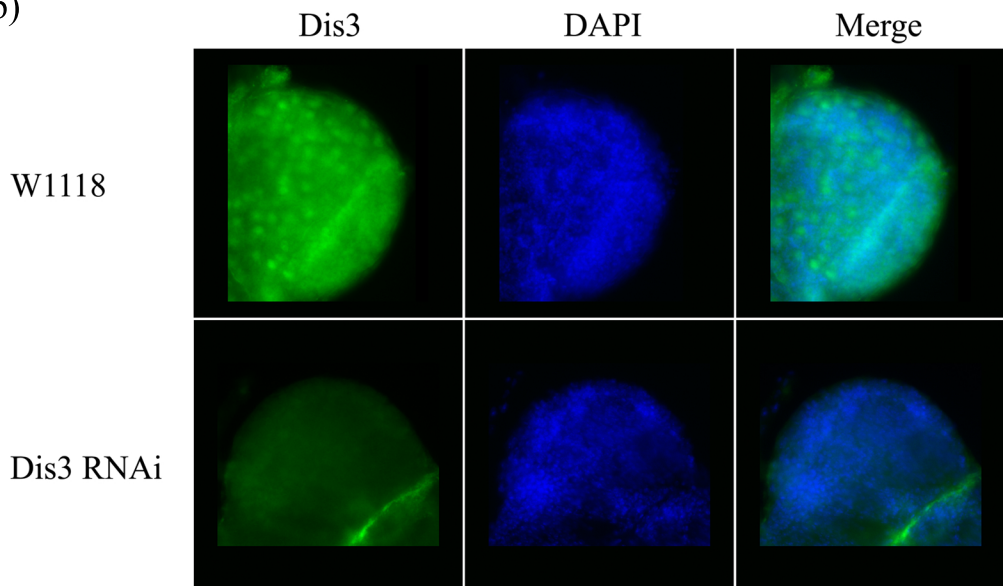

Supplement: Additional file 1 — Figure S1. Dis3 depletion has little or no effect on Rrp6 localization in the fly brain. (a) Wild-type w1118 and Dis3 RNAi brains were dissected and stained with anti-Dis3 (green) and anti-Rrp6 (red) antibodies. Nuclei were stained with DAPI (blue). (b) Close-up visualization of anti-Dis3 antibody and DAPI staining of brains. [file 1471-2164-13-359-S1.pdf]

(a)

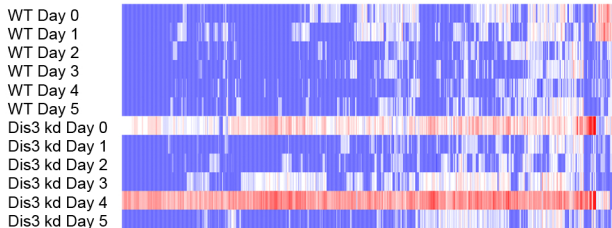

(b)

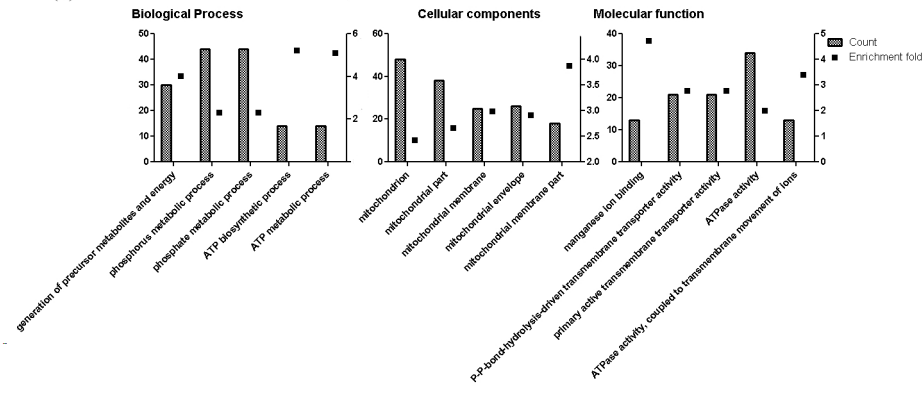

Supplement: Additional file 4 — Figure S2. A subset of mitochondria- and nucleotide-related RNAs highly up-regulated in day 0 and day 4 Dis3KD flies. (a) Comparative heatmap shows the 850 up-regulated RNAs in the Dis3KD flies. (b) GO analysis of the 850 increased transcripts reveals the enriched subset. [file 1471-2164-13-359-S4.pdf]

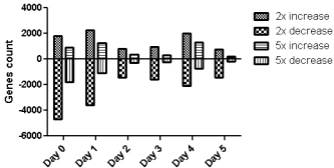

Supplement: Additional file 7 — Figure S3. Detail fold change analysis. 2 fold- and 5 fold-increased and decreased RNAs in Dis3KD samples in all 6 developmental stages. This figure complements Figure 3c. [file 1471-2164-13-359-S7.pdf]

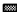 % Count in Selection  
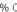 % Count in Total  
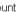 fold enrichment

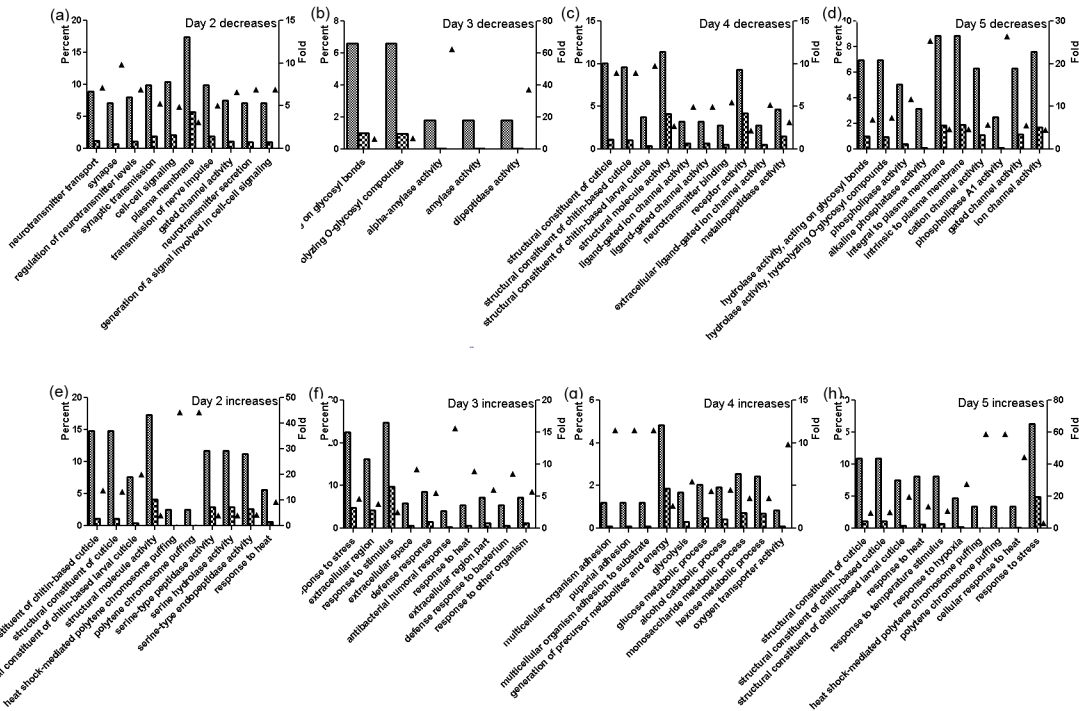

Supplement: Additional file 8 — Figure S4. Gene ontology enrichment analyses of 5 fold-increased and -decreased RNAs for the later developmental time points. Bars depict the percentage of RNAs in selected GO term; black triangles represent fold enrichment, plotted on the right Y axis and calculated by dividing the % count in selection by % count in total. GO terms are sorted by P-value and top 10 were picked at each time point. This figure complements Figure 4. [file 1471-2164-13-359-S8.pdf]

Day 0

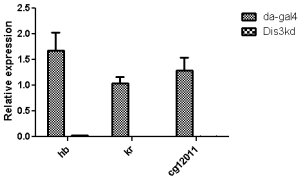

Supplement: Additional file 10 — Figure S5. Transcriptomic changes observed in the Dis3KD flies are not detected in the daughterless-Gal4 early embryos. Expression of hunchback (hb), Krüppel (kr), and CG12011 was assessed by qRT-PCR. [file 1471-2164-13-359-S10.pdf]

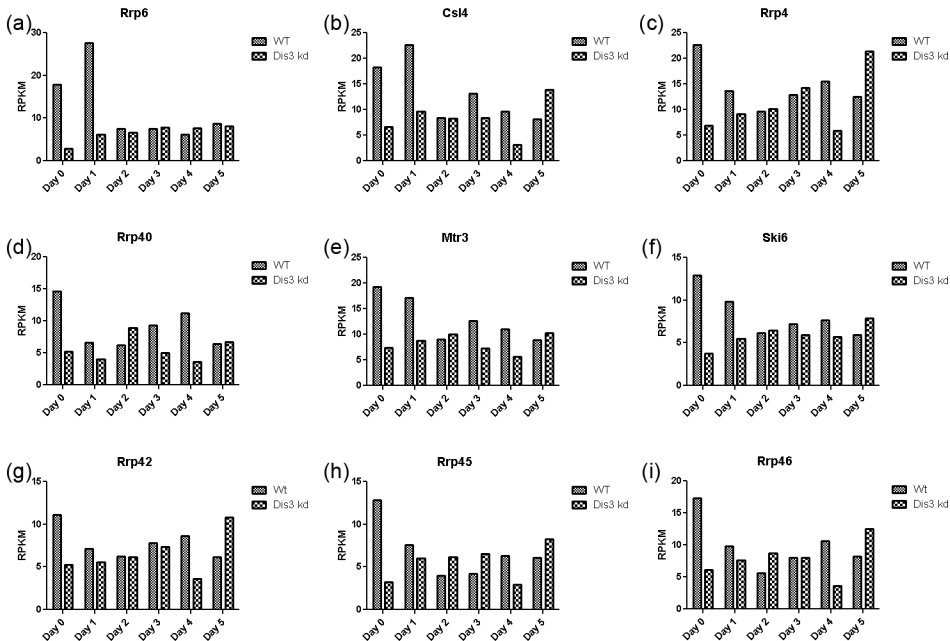

Supplement: Additional file 11 — Figure S6. Relative expression level of Rrp6 and exosome subunit RNAs in wild type and Dis3KD flies over developmental time course. (a) Rrp6, (b) Csl4, (c) Rrp4, (d) Rrp40, (e) Mtr3, (f) Ski6, (g) Rrp46, (h) Rrp45, and (i) Rrp46. [file 1471-2164-13-359-S11.pdf]
